# Do academic advising and levels of support affect nursing students' mental health? A cross‐sectional study

**DOI:** 10.1111/ijn.13267

**Published:** 2024-05-30

**Authors:** Abeer Selim, Nashwa Ibrahim, Shaimaa Awad, Ebtsam Salama, Abeer Omar

**Affiliations:** ^1^ Faculty of Nursing, Psychiatric and Mental Health Nursing Department Mansoura University Mansoura Egypt; ^2^ Mental Health Nursing Department, Faculty of Nursing The British University in Egypt (BUE) Cairo, Shorouk City Egypt; ^3^ Trent/Fleming School of Nursing Trent University Peterborough Ontario Canada

**Keywords:** counselling, mental disorders, nursing, social support, students

## Abstract

**Aim:**

The current study aimed to identify the association between social support, academic advising and mental health disorders among nursing students.

**Background:**

Stress and workload can trigger multiple mental health disorders, especially for nursing students. Thus, academic advising and counselling help support students with academic and mental health problems.

**Design:**

This cross‐sectional study utilized online questionnaires in Egypt and Saudi Arabia.

**Methods:**

Multidimensional Scale of Perceived Social Support (MSPSS), Patient Health Questionnaire (PHQ‐4) and the Student Academic Advising and Counseling Survey (SAACS) were utilized to measure social support, depression and anxiety and evaluation of academic advising and counselling services, respectively.

**Results:**

The study included 1134 nursing students (mean age of 20.3 years). Students with higher academic advising satisfaction were 37% less likely to experience depression (OR 0.63, 95% CI 0.46–0.85) and mental disorders (OR 0.68, 95% CI 0.50–0.94). Moderate family social support was associated with lower depression (OR 0.58, 95% CI 0.37–0.93) and mental disorders (OR 0.55, 95% CI 0.33–0.92).

**Conclusion:**

Academic advising and social support can mitigate mental health disorders among nursing students. These findings will help nurses and post‐secondary providers develop strategies to support nursing students during difficult times.

## Summary statement

What is already known about this topic?

- There is a lack of research on academic advising and its measurement among nursing students.

What does this paper add?

- The current study findings add to the body of knowledge on how academic advising and levels of support affect students' experience of depression and other mental health disorders.

The implication of this paper:

- The findings of the current study will help post‐secondary administrators and stakeholders improve academic advising and counselling and understand the effect of support levels on nursing students' mental health.

## 1 INTRODUCTION

Health profession students, particularly nursing and medical students, reported higher academic stress levels than other departments (Brown et al., 2016; Goff, 2011; van der Walt et al., 2019). Stress can negatively affect nursing students' performance by triggering numerous mental health issues such as sleep disorders, substance use, anxiety and depression (Beiter et al., 2015; Dalky & Gharaibeh, 2019; Pulido‐Criollo et al., 2018). A systematic review reported that depression (52%), fear (41%), anxiety (32%), stress (30%) and sleep disturbances (27%) are the major mental health problems among nursing students (Mulyadi et al., 2021). In addition, 94% of students feel overwhelmed, and 44% feel depressed (American College Health Association, 2018). Globally, depression and anxiety are found to alter students' quality of life in terms of morbidity and social dysfunction, in addition to lower academic performance (American College Health Association, 2018; World Health Organization, 2022a). Although there are effective treatments and prevention strategies for mental diseases, most people who experience mental disorders lack access to them, mainly due to stigma (World Health Organization, 2022a).

Further, the COVID‐19 pandemic has added to stressors on nursing students, contributing to increased mental health disorders (Alsolais et al., 2021; Mulyadi et al., 2021). Lockdown and changing teaching modalities to online classes contributed to increased stressors and lower life satisfaction (Dewart et al., 2020; Gómez‐García et al., 2022). Furthermore, in a Japanese study, the prevalence of depression and anxiety was significantly higher among nursing students during the pandemic (Sakai et al., 2022). The lockdown during COVID‐19 also affected students' social adaptability and networking. University students experienced social isolation, disrupted social services and coping strategies (Appleby et al., 2022).

## 2 BACKGROUND

Social support plays a significant role in the mental health of different populations, including families, university students, workers, older adults and vulnerable populations (Alegría et al., 2018; Hefner & Eisenberg, 2009; Velten et al., 2018). Also, researchers recommend paying more attention to examining the relationship between social support and mental health in high‐risk populations using valid and reliable tools (Harandi et al., 2017). Karaca et al. (2019) reported that social support is a protective factor that promotes mental health among nursing students. Consequently, lack of social support is associated with poor mental health outcomes, recovery and social functioning (Alegría et al., 2018; Hefner & Eisenberg, 2009; Velten et al., 2018). A strong association exists between depression and a lack of social support. Wang et al. (2018) found an association between perceived social support and outcomes in severe mental disorders such as schizophrenia, bipolar and anxiety (Wang et al., 2018). Ghafari et al. (2021) found that medical students with higher social support reported lower scores of mental health issues. In addition, increased perceived social support was associated with mental well‐being and general health (Cobo‐Rendón et al., 2020). However, mental disorders in the light of social support are not well‐studied among nursing students in Egypt and Saudi Arabia.

Academic advising and counselling are significant supportive services that university students need during their academic life. Academic advising and counselling services help screen and support students with academic and mental health problems (Selim et al., 2023). Academic advising serves as a front‐line shield against stressors facing university students (Holland et al., 2020). A recent systematic review concluded that research on nursing students' academic advising is still limited (Chan et al., 2019). Therefore, the current study was conducted to examine the role of types of social support and academic advising in predicting mental health disorders among nursing students in Egypt and Saudi Arabia.

## 3 METHODS

### 3.1 Aims

Examining the role of academic advising and the levels of support for nursing students in Middle Eastern and Arabian countries is limited in research. Therefore, the present study asks the following questions: (1) What are the mental health disorders reported by nursing students? (2) What is the level of social support and mental disorders among nursing students? (3) What is the association between levels of social support and mental health disorders among nursing students? and (4) What is the association between satisfaction with academic counselling and mental health disorders among nursing students? The senior investigator registered the current study using Open Science Framework (OSF) to increase the rigour and transparency of the current methods.

### 3.2 Design

The present study utilized a cross‐sectional design to collect data about mental health disorders, perceived social support levels and academic advising evaluation. In addition, the researchers utilized a correlational approach to determine the predictors of mental health disorders.

### 3.3 Participants

Study participants were recruited from undergraduate nursing students who gave voluntary informed consent and enrolled in years one to four. Exclusion criteria included postgraduate students and those who ignored participation. The study investigators did not exclude students with mental health disorders since many nursing students go underdiagnosed, and few seek mental health services (Morton, 2022). Also, some students diagnosed with mental health issues tend not to comply with treatment plans and follow‐up. The sample size of over 1000 participants was selected for the primary published study of tool validation of academic advising (Selim et al., 2023). This sample size is highly recommended for minimizing error and achieving scale construct validity (Boateng et al., 2018; Comrey & Lee, 1992). An online survey was conducted at two nursing colleges in Egypt and Saudi Arabia, found on the following link (https://forms.office.com/Pages/ResponsePage.aspx?id=GgHvTxXscUGykxsQvFL9oWKi88vR13pFi7oQh7sjvBNUOVIwT1BMRURNWjU5VFlUR0JFTTlONFI2My4u). Out of 3524 nursing students (2974 Egyptians and 550 Saudi Arabians), a convenient sample of 1134 students was selected. The study has a response rate of 32.2%.

### 3.4 Data collection

Data were collected through an online survey after the two sites' approval by Institutional Review Board (IRB). All undergraduate nursing students enrolled in the BSN programme received the online survey link via emails and social communication applications (WhatsApp© groups; 2024 WhatsApp©). In addition, the researchers sent weekly reminders through emails to enhance data collection. The same data collection steps were followed at both sites and took 2 months, beginning in March and ending in April 2021.

Socio‐demographic data included students' age, gender, academic year, marital status, number of children, residence, GPA, employment, leisure activities, physical and mental problems/disorders, frequency of individual meetings with the adviser and preferred way of communication with academic advisors. The study used the Multidimensional Scale of Perceived Social Support (MSPSS), developed by Zimet et al. in 1988, to assess social support among nursing students (Zimet et al., 1988). This tool assesses perceptions of social support adequacy from three specific sources: family, friends and significant other, and it consists of 12 items. Each item was rated on a 7‐point Likert scale ranging from *very strongly disagree* (1 point) to *very strongly agree* (7 points). Lower scores indicate lower social support. For example, a mean total scale score of 1–2.9 indicates low support, a score of 3–5 indicates moderate support and a score from 5.1 to 7 is considered high support. The items of the Arabic version of MSPSS are rated on a 5‐point Likert scale (Merhi & Kazarian, 2012).

The Patient Health Questionnaire for Depression and Anxiety (PHQ‐4) was used to measure depression and anxiety. The PHQ‐4 tool was developed by Kroenke et al. (2009) as an ultra‐brief screener for depression and anxiety. The items of PHQ‐2 (Kroenke et al., 2003) that assess depressive disorders and the two core criteria of GAD (Kroenke et al., 2007) that measure anxiety disorders were combined to compose a four‐item scale (PHQ‐4). The total score ranges from 0 to 12. Scores are rated as normal (0–2), mild (3–5), moderate (6–8) and severe (9–12) (Kroenke et al., 2009). The PHQ‐4 is a reliable and valid tool of 0.81 Cronbach's alpha. Corrected item‐total correlations for PHQ‐4 were between *r* = 0.66 and *r* = 0.80. PHQ‐4 area under the curve (AUC) values were 0.835 and 0.787, respectively, for anxiety and depression (Khubchandani et al., 2016).

The Student Academic Advising and Counseling Survey (SAACS) was developed and validated by researchers to evaluate academic advising and counselling services (Selim et al., 2023). The survey comprised 18 items; Items 1–17 are rated on a five‐point Likert (5 = *strongly agree*, and *strongly disagree* = 1). However, Item 18 is rated as (*strongly satisfied* = 5 to *strongly dissatisfied* = 1). The SAACS showed excellent content validity of 0.989 and universal agreement S‐CVI/UA scores of 0.944. The SAACS internal consistency using Cronbach's alpha was 0.97 (95% CI: 0.966–0.972).

### 3.5 Ethical considerations

IRB approval was secured from the two sites before data collection. IRB was obtained from the National Committee of Bioethics at King Abdullah International Medical Research Center (Registration number: H‐01‐R‐005) in Saudi Arabia and the Faculty of Nursing Research Ethics Committee (Registration number: 0220) in Egypt. The study followed the ethical principles of the World Medical Association Declaration of Helsinki.

No identifiers or personal information was collected or stored. Anonymous data were collected from students using an online survey. The informed consent and a statement explaining the purpose of the study and the participant rights' regarding voluntary and confidential participation were included in the email invitation to students. The participants were assured that data would be used only for research purposes, and their privacy and confidentiality were protected.

### 3.6 Data analysis

The study investigators analysed the data using software version 4.1.3 (The R Project for Statistical Computing, Vienna, Austria). The univariate analysis summarized continuous data using means and standard deviations and categorical variables using frequencies and percentages. The investigators chose three outcomes (depression, anxiety and mental disorders) to develop bivariate and multivariate analyses in measuring associated predictors. The mental disorder was defined by having a score of ≥3 on either of the PHQ‐2 depression and the PHQ‐2 anxiety subscales of the PHQ‐4.

The study investigators included potential predictors of mental health disorders for nursing students in three multiple logistic regression analyses, one for each of the three chosen mental health outcomes (anxiety, depression and mental disorders). The potential predictors included the socio‐demographic characteristics (country, age, gender, marital status, having children, residence type and employment), frequency of leisure activities, self‐reported mental and physical disorders, perceived social support (measured by the MSPSS scale), current GPA, academic year and satisfaction with academic advising services. The included predictors were tested for multicollinearity (generalized variance inflation factor ranged from 1.1 to 3.8) and for influential outliers (all Cook's distance values < 0.5). Results of bivariate logistic regression analyses (unadjusted OR) were reported along with multiple logistic regression analyses (adjusted OR).

## 4 RESULTS

### 4.1 Participants characteristics

Table 1 shows the details of the participants' characteristics. This study included 1134 nursing students (64.3% from Egypt and 35.7% from Saudi Arabia), with a mean age of 20.3 ± 1.4 years, and 81.9% of whom were females. The students were equally sampled from the four academic years. In addition, the results showed that most of the students (83%) were not engaged in frequent leisure activities per week. On the other hand, almost three‐quarters (74%) reported satisfaction with academic advising. Table 2 displays the self‐reported mental and physical disorders. We found the most reported mental health disorders were insomnia (18.3%), depression (10.3%), eating disorders (7.5%) and anxiety (6.8%). Over two‐thirds of students reported at least one physical disorder. About one‐fifth of self‐reported disorders were anaemia and headache.

**TABLE 1:** Nursing Students' characteristics.

Variables	Total (*n* = 1134)	
	*n*	%
Age in years (Mean ± SD)	20.3 ± 1.4	
Country		
Egypt	729	64.29
Saudi Arabia	405	35.71
Gender		
Male	205	18.1
Female	929	81.9
Academic year		
First	275	24.3
Second	308	27.2
Third	296	26.1
Fourth	255	22.5
Marital status		
Single	1084	95.6
Married	49	4.3
Divorced	1	0.1
Children (yes)	46	4.1
Residence		
Alone	21	1.9
With families or relatives	1064	93.8
With classroom mates	49	4.3
Current GPA (Mean ± SD)^a^	3.5 ± 0.6	
Employment		
Unemployed	1047	92.3
Part‐time	78	6.9
Full‐time	9	0.8
Frequency of leisure activities per week		
None	224	21.5
Once/rarely	284	25.0
Sometimes	413	36.4
Often	142	12.5
Always	51	4.5
Satisfaction with academic advising		
Yes	841	74.2
No	293	25.8

^a^ GPA was missing for 71 respondents.

Statistically significant.

**TABLE 2:** Nursing students' self‐reported mental and physical disorders.

Variables	Total (*n* = 1134)	
	*n*	%
Self‐reported mental disorders	326	28.7
Insomnia	207	18.3
Depression	117	10.3
Mood disorders	16	1.4
Anxiety	77	6.8
Eating disorders	85	7.5
Suicidal thoughts	26	2.3
Self‐harm behaviours	21	1.9
Obsessive‐compulsive disorders	26	2.3
Self‐reported physical disorders	432	38.1
Anaemia	230	20.3
Headache	231	20.4
Gastrointestinal disease	93	8.2
Cardiovascular disease	13	1.1
Respiratory disease	37	3.3
Endocrine disorder	20	1.8

### 4.2 Level of perceived social support

Table 3 presents a summary of the results obtained from the Multidimensional Scale of Perceived Social Support Questionnaire (MSPSS). Our analysis revealed that none of the students reported high levels of perceived social support. Instead, they reported either moderate or low levels of social support from family, friends and others. The majority of the students reported moderate perceived support in each dimension of the MSPSS scale.

**TABLE 3:** Nursing Students' scores on the Multidimensional Scale of Perceived Social Support Questionnaire (MSPSS).

Variable	Mean ± SD	Total (*n* = 1134)	
		*n*	%
Others support questions			
1. There is a special person who is around when I am in need.	3.8 ± 1.0		
2. There is a special person with whom I can share joys and sorrows.	3.8 ± 1.1		
5. I have a special person who is a real source of comfort to me.	3.9 ± 1.1		
10. There is a special person in my life who cares about my feelings.	3.8 ± 1.2		
Others support score^a^	3.8 ± 0.9		
Low		167	14.7
Moderate		967	85.3
Family support questions			
3. My family really tries to help me.	4.2 ± 0.9		
4. I get the emotional help & support need from my family.	4.1 ± 1.0		
8. I can talk about my problems with my family.	3.4 ± 1.3		
11. My family is willing to help me make decisions.	3.8 ± 1.0		
Family support score^a^	3.9 ± 0.9		
Low		159	14.0
Moderate		975	86.0
Friends support questions			
6. My friends really try to help me	3.9 ± 1.0		
7. Can count on my friends when things go wrong.	3.7 ± 1.1		
9. I have friends with whom I can share my joys and sorrows.	3.7 ± 1.1		
12. I can talk about my problems with my friends.	3.6 ± 1.1		
Friends support score^a^	3.7 ± 1.0		
Low		200	17.6
Moderate		934	82.4
Total MSPSS score^a^	3.8 ± 0.8		
Low		168	14.8
Moderate		966	85.2

^a^ Low support scores from 1 to 2.9; moderate support from 3 to 5; and high support from 5.1 to 7.

### 4.3 Mental health disorders

Table 4 illustrates the students' responses to the patient health questionnaire (PHQ‐4). We found that over half (52%) of the students were suffering from a mental disorder on PHQ‐4. Of those, 41.9% had anxiety, and 40.6% had depression.

**TABLE 4:** Mental disorders among nursing students based on their score on PHQ‐4.

Variable	Mean ± SD	*n*	%
PHQ‐2 anxiety			
1. Feeling nervous, anxious or on edge	1.4 ± 1.0		
2. Not being able to stop or control worrying	1.1 ± 0.9		
PHQ‐2 anxiety score	2.5 ± 1.7		
Anxiety^a^		475	41.9
PHQ‐2 depression			
3. Little interest or pleasure in doing things	1.3 ± 1.0		
4. Feeling down, depressed or hopeless	1.3 ± 1.0		
PHQ‐2 depression score	2.6 ± 1.8		
Depression^b^		460	40.6
PHQ‐4 score	5.1 ± 3.2		
Mental disorders^c^		596	52.6

^a^ PHQ‐2 anxiety score ≥ 3.

^b^ PHQ‐2 depression score ≥ 3.

^c^ Mental disordersare defined as having a score ≥3 on either of the PHQ‐2 depression or PHQ‐2 anxiety subscales of the PHQ‐4.

### 4.4 Predictors of mental health disorders

#### 4.4.1 Unadjusted analysis

Table 5 includes the results of the bivariate and multiple logistic regression analyses. In the bivariate analyses, factors significantly associated with lower odds of mental disorders are male gender, having children, higher perceived social support and satisfaction with academic advising. Males were less likely to be depressed (OR = 0.7, 95% CI [0.48, 0.91]), have anxiety (OR = 0.5, 95% CI [0.39, 0.75]) or mental disorders (OR = 0.6, 95% CI [0.42, 0.77]). Participants who had children were less associated with depression (OR = 0.5, 95% CI [0.26, 0.98]). Those with moderate perceived social support from friends, family and others were not associated with depression, anxiety or mental disorders (the same for every three dimensions). Participants who were satisfied with academic advising were less depressed (OR: 0.46, 95% CI [0.35, 0.66]), had anxiety (OR = 0.56, 95% CI [0.43, 0.73]) or mental disorders (OR = 0.49, 95% CI [0.37, 0.64]).

**TABLE 5:** Bivariate and multiple logistic regression analysis of predictors associated with depression, anxiety and mental disorder.

Predictors	Depression		Anxiety		Mental Disorders^a^	
	Unadjusted OR (95% CI)	Adjusted OR (95% CI)	Unadjusted OR (95% CI)	Adjusted OR (95% CI)	Unadjusted OR (95% CI)	Adjusted OR (95% CI)
Country (Saudi Arabia vs. Egypt)	1.15 (0.90, 1.47)	1.22 (0.89, 1.65)	1.18 (0.92, 1.50)	1.21 (0.89, 1.64)	1.13 (0.89, 1.45)	1.19 (0.88, 1.62)
Age in years	1.06 (0.98, 1.15)	1.06 (0.90, 1.25)	1.08 (0.99, 1.17)	0.94 (0.80, 1.11)	1.06 (0.97, 1.14)	0.96 (0.81, 1.13)
Gender (male)	0.66* (0.48, 0.91)	0.81 (0.53, 1.23)	0.54*** (0.39, 0.75)	0.66 (0.44, 1.01)	0.57*** (0.42, 0.77)	0.67* (0.44, 1.00)
Academic year (reference to first)						
Second	1.16 (0.83, 1.62)	1.03 (0.67, 1.59)	1.40* (1.00, 1.96)	1.39 (0.90, 2.15)	1.17 (0.85, 1.63)	1.11 (0.72, 1.70)
Third	1.33 (0.95, 1.86)	0.91 (0.53, 1.56)	1.75** (1.25, 2.45)	1.57 (0.91, 2.70)	1.64** (1.17, 2.28)	1.41 (0.83, 2.40)
Fourth	1.15 (0.81, 1.63)	0.94 (0.47, 1.86)	1.37 (0.96, 1.95)	1.54 (0.77, 3.08)	1.20 (0.85, 1.68)	1.28 (0.65, 2.51)
Marital status (single or divorced vs. married)	1.74 (0.93, 3.28)	1.48 (0.67, 3.26)	1.25 (0.69, 2.27)	1.03 (0.48, 2.20)	1.64 (0.92, 2.94)	1.37 (0.64, 2.91)
Children (yes)	0.50* (0.26, 0.98)	0.56 (0.25, 1.30)	0.81 (0.44, 1.49)	0.83 (0.38, 1.81)	0.62 (0.34, 1.13)	0.74 (0.34, 1.60)
Residence (reference to living alone)						
With families or relatives	0.62 (0.26, 1.48)	0.49 (0.19, 1.30)	1.15 (0.47, 2.81)	0.97 (0.37, 2.55)	1.00 (0.42, 2.38)	0.80 (0.31, 2.09)
With classroom mates	0.48 (0.17, 1.36)	0.46 (0.15, 1.41)	1.69 (0.60, 4.81)	1.91 (0.62, 5.85)	1.12 (0.40, 3.11)	1.15 (0.38, 3.47)
Current GPA	0.94 (0.76, 1.15)	0.93 (0.73, 1.19)	1.01 (0.82, 1.24)	0.98 (0.76, 1.25)	0.94 (0.77, 1.16)	0.90 (0.71, 1.15)
Employment (reference to full‐time)						
Unemployed	2.38 (0.49, 11.52)	1.70 (0.33, 8.84)	5.60 (0.70, 44.92)	4.55 (0.52, 40.02)	3.80 (0.79, 18.38)	3.05 (0.58, 16.18)
Part‐time	2.70 (0.53, 13.86)	1.79 (0.32, 9.86)	9.83* (1.17, 82.39)	8.32 (0.90, 77.16)	5.91* (1.15, 30.40)	4.83 (0.85, 27.41)
Frequency of leisure activities per week (reference to always)						
Never	4.17*** (2.00, 8.70)	3.07** (1.38, 6.80)	2.25* (1.17, 4.32)	1.44 (0.69, 3.02)	2.73** (1.46, 5.12)	1.80 (0.89, 3.65)
Sometimes	1.90 (0.93, 3.92)	1.74 (0.80, 3.78)	1.35 (0.72, 2.56)	1.09 (0.54, 2.22)	1.58 (0.86, 2.89)	1.31 (0.67, 2.58)
Often	2.37* (1.10, 5.12)	2.34* (1.02, 5.33)	1.23 (0.61, 2.46)	1.03 (0.48, 2.23)	1.46 (0.75, 2.84)	1.28 (0.61, 2.66)
Once/rarely	4.22*** (2.03, 8.75)	3.52** (1.60, 7.75)	2.50** (1.31, 4.78)	1.77 (0.85, 3.65)	3.08*** (1.65, 5.74)	2.26* (1.13, 4.55)
Self‐reported one or more mental disorders	4.20*** (3.20, 5.51)	3.00*** (2.19, 4.10)	4.87*** (3.69, 6.43)	3.26*** (2.38, 4.45)	5.03*** (3.73, 6.79) ***	3.17*** (2.27, 4.43)
Self‐reported one or more physical disorders	2.06*** (1.61, 2.63)	1.20 (0.89, 1.62)	2.78*** (2.17, 3.56)	1.62** (1.21, 2.17)	2.78*** (2.16, 3.57)	1.64** (1.22, 2.20)
Satisfied with academic advising	0.46*** (0.35, 0.60)	0.63 ** (0.46, 0.85)	0.56 *** (0.43, 0.73)	0.77 (0.57, 1.05)	0.49 *** (0.37, 0.64)	0.68 * (0.50, 0.94)
Perceived social support based on MDSPSS						
Total social support (moderate vs. low)	0.29*** (0.21, 0.41)	0.92 (0.48, 1.77)	0.37*** (0.27, 0.52)	0.90 (0.46, 1.74)	0.32*** (0.22, 0.46)	1.07 (0.54, 2.13)
Family support (moderate vs. low)	0.26*** (0.18, 0.37)	0.58* (0.37, 0.93)	0.34*** (0.24, 0.48)	0.73 (0.45, 1.16)	0.25*** (0.17, 0.38)	0.55* (0.33, 0.92)
Friends support (moderate vs. low)	0.42*** (0.31, 0.58)	0.88 (0.57, 1.36)	0.51*** (0.38, 0.70)	0.94 (0.60, 1.46)	0.44*** (0.32, 0.61)	0.82 (0.52, 1.30)
Others support (moderate vs. low)	0.30*** (0.21, 0.43)	0.59 (0.35, 1.02)	0.37*** (0.26, 0.51)	0.61 (0.36, 1.06)	0.31*** (0.21, 0.45)	0.55* (0.31, 0.97)

^a^ Mental disorders are defined as having a score ≥3 on either of the PHQ‐2 depression or PHQ‐2 anxiety subscales of the PHQ‐4.

* *p* < 0.05,

** *p* < 0.01, and

*** *p* < 0.001.

In contrast, factors associated with higher odds of anxiety, mental disorders and depression in the bivariate analyses included academic year, part‐time employment, lower frequencies of leisure activities and those who reported at least one physical disorder. Second and third‐year students (OR = 1.4, 95% CI [1.00, 1.96]; and OR = 1.8, 95% CI [1.25, 2.45], respectively), part‐time employees (OR = 9.83, 95% CI [1.17, 82.39]) and those who reported at least one physical disorder (OR = 2.78, 95% CI [2.17, 3.56]) were associated with higher levels of anxiety. As regards mental disorders, third year (OR = 1.6, 95% CI [1.17, 2.28]); part‐time employment (OR = 5.91, 95% CI [1.15, 30.4]); lower frequencies of leisure activities as never, once or rarely per week; and self‐reporting of at least one physical disorder (OR = 2.78, 95% CI [2.16, 3.57]) had higher odds of mental disorders. Regarding depression, rarely engagement (OR = 4.22, 95% CI [2.03, 8.75]) and self‐reporting of at least one physical disorder (OR = 2.06, 95% CI [1.61, 2.63]) were associated with the highest odds of depression.

#### 4.4.2 Adjusted analysis

Table 5 also includes the logistic regression results for the 3 outcomes (depression, anxiety, mental disorders). None of the socio‐demographic factors and total social support were significant predictors of the three outcomes. Those who perceived higher social support from families were less likely to experience depression (OR = 0.58, 95% CI [0.37, 0.93]) and mental disorders (OR = 0.55, 95% CI [0.33, 0.92]). Students who perceived other social support sources were less likely to have mental disorders (OR = 0.55, 95% CI [0.31, 0.97]). Finally, students who reported higher satisfaction with academic advising were 37% less likely to experience depression (OR = 0.63, 95% CI [0.46, 0.85]) and mental disorders (OR = 0.68, 95% CI [0.50, 0.94]). In contrast, predictors significantly associated with higher odds of mental disorders included the following: students who never had leisure activities per week were 3 times more likely to experience depression (OR = 3.07, 95% CI [1.38, 6.80]); those with once/rarely were more likely to be depressed (OR = 3.52, 95% CI [1.60, 7.75]) or have mental disorders (OR = 2.26, 95% CI [1.13, 4.55]). In addition, students who reported at least one physical disorder were more likely to experience anxiety (OR = 1.62, 95% CI [1.21, 2.17]) and mental disorders (OR = 1.64, 95% CI [1.22, 2.20]).

## 5 DISCUSSION

The study findings show that more than half of the nursing students suffered from mental disorders, either anxiety and/or depression. Perceived social support ranged from low to moderate in total and in all dimensions among nursing students. Adjusted analysis indicates that perceived family and others' social support and satisfaction with academic advising were significantly associated with lower odds of mental disorders included. In contrast, predictors significantly associated with higher odds of mental disorders were lower frequencies of leisure activities per week, self‐reporting of at least one mental disorder and self‐reporting of at least one physical disorder.

Social support plays a significant role in facing stress and correlates significantly with resilience among nursing students (Ozsaban et al., 2019). Regarding gender and in support of our results, it was reported that among depressed participants, males scored much higher on the perceived social support scale than females did (Soman et al., 2016). Zhou et al. (2022) reported that social support promotes mental health among nursing students (Zhou et al., 2022). These results explain why nursing students in our study did not show high levels of social support and were experiencing high levels of anxiety and depression. In contrast, a Nepalese study reported that nursing students had high levels of stress, anxiety and depression, as well as high levels of social support (Samson, 2020).

Leisure activities significantly improve mental health and tend to decrease depression and anxiety across different populations (Chen et al., 2022; Goodman et al., 2017; Nagata et al., 2020). Additionally, signs of depression and anxiety appear to be highly correlated with both physical inactivity and sedentary behaviour (Bélair et al., 2018). Similar to our results, Cheung and colleagues (2016) found that lack of leisure time and reporting mental illness were strongly correlated with anxiety and depression among nursing students (Cheung et al., 2016). The present study shows the body–mind link because reporting physical disorders predicted mental disorders. Physical disorders are strongly associated with depression and anxiety and vice versa (Strine et al., 2004).

Although this study was conducted while COVID‐19 restrictions were reduced and the students resumed their clinical training onsite, the pandemic might affect their mental health and worsen their anxiety and depression levels. For example, earning during the COVID‐19 pandemic has heightened university students' levels of stress, anxiety and depression (Fawaz & Samaha, 2021). The COVID‐19 pandemic significantly increased the number of persons who experienced anxiety and depressive disorders in 2020 (World Health Organization, 2022b).

This study attempted to address the gap in research by examining the satisfaction with academic advising's association with mental health disorders in nursing students. Recruiting a large sample size is considered one of the study's strengths. The study also utilized valid and reliable tools to measure the variables of interest.

### 5.1 Study limitations

Using a cross‐sectional design might limit the generalizability of the current results to only included countries. An additional limitation is the lack of conducting qualitative interviews to examine students' in‐depth insights regarding the relationship between social support and mental health disorders in Arabian countries.

## 6 CONCLUSION

Mental disorders such as depression and anxiety are common among nursing students who experience middle and lower levels of social support, although most of them live with their families. Family and others' support as well as satisfaction with academic advising were significant predictors of lower levels of mental disorders among nursing students. Therefore, counselling and guidance by nursing educators/faculty are highly recommended as a defence in the battle of stress and mental disorders that students face during their educational journey. The study investigators recommend conducting interventions for nursing students in the Middle East to mitigate stressors and enhance nursing students' social support to boost their mental health.

## AUTHORSHIP STATEMENT

All listed authors meet the authorship criteria and that all authors are in agreement with the content of the manuscript. All authors have participated in conceptualization and designing the study. AS led the data collection and draft writing. SA and ES have collected the data. AS, NI and AO drafted the manuscript. AO supervised the writing, methodology and editing and revised the data analysis.

## CONFLICT OF INTEREST STATEMENT

All authors confirm that no financial or personal relationships can lead to a conflict of interest regarding this study.

## Data Availability

The data that support the findings of this study are available from the corresponding author upon reasonable request.
